# Disparities in Distress Symptoms Among Cancer Inpatients, Outpatients and Relatives Through Introducing and Evaluating Digital Distress Screening

**DOI:** 10.1002/pon.70401

**Published:** 2026-02-14

**Authors:** Tana Dornbrach, Madeleine Volz, Thomas Seufferlein, Hans Kestler, Klaus Hönig

**Affiliations:** ^1^ Department of Psychosomatic Medicine and Psychotherapy University Clinic of Ulm Albert‐Einstein‐Allee Germany; ^2^ Department of Internal Medicine University Clinic of Ulm Albert‐Einstein‐Allee Germany; ^3^ Department of Medical Systems Biology University of Ulm Albert‐Einstein‐Allee Germany

**Keywords:** cancer, digital acceptance, digitalisation, distress, oncology, psycho‐oncology, relatives, screening, usability

## Abstract

**Background & Aims:**

Cancer treatments and survival rates have significantly improved, yet distress in patients and their relatives remains overlooked, leaving them with needs unmet. One contributing factor is inadequate screening, which could be improved by digitalisation. This study examined differences in distress among outpatients, inpatients, and their relatives, along with acceptance and usability of digital screening tools.

**Methods:**

149 participants including relatives, outpatients, and inpatients were randomised using established analogue screening versus digital screening with text‐based instructions or digital screening with video‐based instructions for the Distress Thermometer and rated their distress. Participants then provided ratings for usability and acceptance of digital screening measures.

**Results:**

Overall distress levels on average were high for all, inpatients, outpatients and relatives. There were no significant differences between groups in overall distress levels. Inpatients without wish for counseling reported greater physical distress and lower psychological and psychosocial distress than inpatients with wish for counseling, who were similar to relatives and outpatients. Participants expressed high overall satisfaction with digital screening measurements. Digital screening with text‐based instructions seems to be superior to analogue screening or video‐based screening, regardless of age and gender, when people have hands‐on experience. Patients and relatives using analogue screening are more skeptical of digital screening, especially elderly and female users.

**Conclusion:**

Our study offered valuable insights into the varying distress levels of inpatients, outpatients, and relatives, which leads to the implication that outpatients and relatives should also be screened closely. The decisive variable was wish for counseling. There is a need for more counseling options for both cancer patients and their relatives. Our findings support the use of digital screening methods for patients and relatives. The hands‐on experience seems to be crucial for a higher acceptance.

## 1 Background

The global increase in oncologic diseases causes a significant enhancement in mental health issues among cancer patients [1]. About 4%–49% of cancer patients report mental disorders, such as major depression. Even more show subclinical distress symptoms such as feelings of sadness and anxiety, pain or fatigue [2] and express a wish for professional psychological support. However, only 38% receive counseling [3]. Fortunately, early screening and intervention can positively impact distress, recovery, and survival chances, as demonstrated by numerous studies [4, 5].

The importance of screening outpatients through counseling or treatment appointments is increasing due to a progressing shift from hospitalisation to day clinic care [6]. A majority of studies show less distress in outpatients due to lack of hospitalisation effects and study design [7, 8]. Other research suggests that outpatients experience higher distress after active treatment [9, 10], while inpatients experience fewer symptoms over hospitalisation duration [11]. Damocles syndrome, PTSD, and finding their way back to everyday life also contribute to distress for survivors, which is often aggravated by expectations of relatives [12]. Previous research indicates that female and younger cancer patients, as well as those with poor social and financial support experience higher distress [13, 14]. According to best practice guidelines, cancer patients should undergo regular structured screening over the course of treatment to detect changes in distress and consultation needs, which is far from reality [15].

One approach to increase the still unsatisfactory screening numbers is the digitalisation of instruments, directly targeting the main reasons: lack of time and manpower [16, 17]. Digitalisation in modern medicine, including oncology care, is beneficial on a structural and organisational level, as well as for treatment [18]. Health information technology (HIT) is increasingly integrated into medical care systems, with patient acceptance and feasibility of digital measures evaluated high or very high [19]. One approach of HIT is electronic patient‐reported outcomes (ePRO/ePROM), which appear as superior, due to decreased time consumption, instantly accessible information, swift follow‐up treatment, environmental outcome, accessibility to disabled individuals, integrating data into digital patient files, and less workload for medical staff [20, 21]. Furthermore, paper printing, management and storage can be eliminated. Depending on available hardware and software, or need of programming platforms/apps, there is the possibility of reduce costs [22]. Acceptance is reported as independent of sex, age, education and distress levels, while feasibility is lower for older patients and patients with lower Karnofsky‐index [23]. Direct comparison between digital and analogue screening shows no difference in scores and diagnostic sensitivity [24].

While digital text‐based screening has continued to increase in recent years, digital video‐based screening is still an exception. Research focused so far mostly on the exploration of declarative knowledge through explanation videos, whereas distress screening is not yet considered an area of application. Educational videos can address the issue of limited human resources for patient instruction, as they improve patient‐doctor communication in clinical settings [25]. While some findings suggest effects highly depend on the design and usability of videos, others found no difference to written explanations [26]. It is therefore questionable whether the additional effort involving in creating an instructional video is really worthwhile.

### 1.1 Purpose

The present study aims to identify possible differences in distress between inpatients, outpatients, and their relatives, representing a broad range of individuals affected by cancer. Furthermore, we want to investigate the usability and acceptance of digital distress screening with digital text‐based and video‐based instruction in cancer patients compared to established analogue measures.

## 2 Methods

### 2.1 Participants

Eligibility was based on being a cancer patient or relative attending an outpatient cancer counseling centre or cancer daycare in Ulm. Patients and relatives did not have to be matched and could participate independently. Outpatients were partly still in active treatment, remission or after remission. We later extended the sample by including cancer inpatients from a general oncology ward of University Clinic Ulm.

### 2.2 Study Design

Recruitment and enrollment took place from 2020 to 2023. This timeline coincides directly with the COVID‐19 pandemic and led to many unusual circumstances, which also influenced the study. For instance, the distinction between which treatments were carried out on an outpatient or inpatient basis depended less on the treatment itself than it normally would, and more on the circumstances of the patient and clinic capacities. Furthermore, patients and relatives were significantly more distressed, as well as medical staff had a massive workload [27].

**FIGURE 1:**
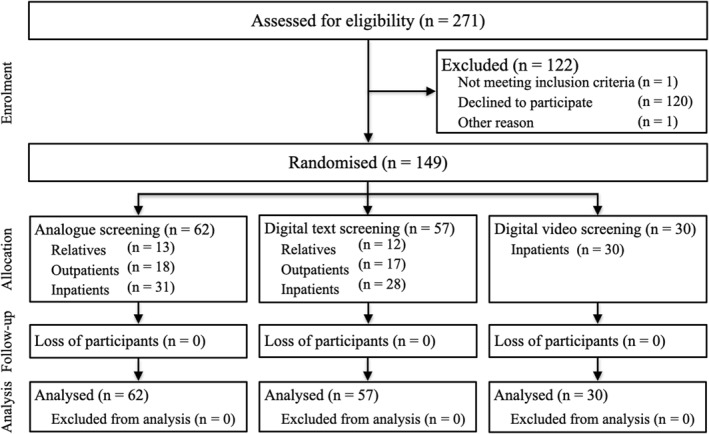
Trial profile.

As part of mandatory screening procedure, newly admitted patients were informed about study objectives and voluntarily participation. Informed consent was obtained. If participation was rejected, no consequences were incurred. Participants were randomised via software in analogue versus digital text screening and completed a sociodemographic questionnaire, followed by the distress screening. Staff remained close‐by, in case of need for assistance. Participants in the analogue screening condition were handed a clipboard with printed screening, while participants in the digital conditions were handed a tablet. In the second trial, we added a digital video screening for inpatients (see Figure 1). Due to copyright reasons and delayed trial start, we could only use video screening for inpatients. Participants read or viewed the screening instructions, rated their distress levels and later evaluated the screening procedure for usability and acceptance. All analogue screening participants were asked for their opinion on using digital features for screening. A priori sample size calculations required 18 people per subgroup for average effect sizes. The procedure required 20–30 min. Support was provided for participants struggling with study procedure, such as explaining how to use or hold the tablet device. However, distress screening had to be done without help to maintain representative conditions. No participant needed excessive support. Analogue screening participants were offered support as well, though no one had any need.

### 2.3 Measures

For the evaluation of a digital distress screening the platform *FeelBack* was developed. *FeelBack* allows the collection of data on distress, using the German version of the *Distress Thermometer (DT)* [28] on smart mobile devices (tablets). The developmental process of the FeelBack app as well as first results of feasibility and acceptance in outpatients and relatives are published in Schobel et al., 2021 [29].

DT was used since it is established in the clinical setting of the University Clinic of Ulm and it is considered the gold‐standard in distress screening in psycho‐oncology [28]. The DT includes items for psychosocial, psychological, and physical distress and a single item for overall distress. To assess usability and acceptance of digital screening, questionnaires were adopted from a previous study in Tübingen [29].

For evaluation ratings, a bipolar Likert‐scale was used, ranging from 1 (negative response/not agreeing) to 10 (positive response/fully agreeing). Symptoms of distress and wish for counseling had dichotomous yes/no answer format. Respective psychometric statistics are reported in Tables 1 and 2.

**TABLE 1:** Distress of relatives and patients.

	Distress *M* (SD)	Psychosocial distress *M* (SD)	Psychological distress *M* (SD)	Physical distress *M* (SD)
Relatives	6.60 (2.35)	1.50 (1.30)^*^ ^b^	5.70 (2.30)^*^ ^b^	1.80 (1.50)^**^ ^b^
Outpatients	6.31 (2.03)	2.00 (1.70)^**^ ^b^	6.60 (3.30)^**^ ^b^	2.70 (1.60)^*^ ^b^
Inpatients	6.16 (2.56)	0.80 (1.20)	4.00 (3.10)	3.90 (2.00)
Inpatients without wish for counseling	5.81 (2.64)	0.70 (1.10)^a^	3.50 (3.00)^a^	3.70 (2.00)^a^
Inpatients with wish for counseling	7.29 (1.93)	1.20 (1.50)^a^	5.40 (3.00)^*^ ^b^	4.30 (2.00)^a^

*Note:* Distress subscales transformed for comparability. a; b = significance code. Groups with different letters differ significantly post‐hoc.

^*^ *p* < 0.05.

^**^ *p* < 0.001.

**TABLE 2:** Usability and digital acceptance of screening varieties.

	Usability *M* (SD)	Digital acceptance *M* (SD)
Analogue screening	8.40 (1.30)^*^ ^b^	4.83 (2.30)^a^
Digital text screening	8.83 (1.40)^a^	7.61 (2.35)^**^ ^b^
Digital video screening	8.31 (1.49)	7.43 (2.13)^**^ ^b^

*Note:* a; b = significance code. Groups with different letters differ significantly post‐hoc

^*^ *p* < 0.05.

^**^ *p* < 0.001.

### 2.4 Statistics

Analysis was performed with IBM SPSS 29 and RStudio. Data preprocessing showed neither substantially nor systematically missing data. Group differences in descriptive statistics were tested. For calculation of the overall digital acceptance (four indicator variables), overall acceptance of the questionnaire (three indicator variables), psychometric statistics for reliability were calculated for overall population and subsamples. McDonald's Omega was ranging between *ω* = 0.54 to *ω* = 0.91 for overall population (see Table A2). Minor values arise from heterogenous items within scales.

## 3 Results

### 3.1 Sample Descriptive

Table A1 displays descriptive statistics for sample variables for each setting. A total of 149 individuals was included. 25 relatives (*M*_*r*_ = 49.04 years; *SD*_*r*_ = 14.80) and 35 outpatients (*M*_*o*_ = 56.54 years; *SD*_*o*_ = 10.32) were recruited at the cancer counseling centre. One participant did not meet inclusion criteria and was excluded respectively. 72% and 77.10% were female, representing a common gender ratio in people seeking support in psycho‐oncology [30]. There were no significant differences between groups in descriptive variables. In our second trial, 89 inpatients (*M*_*i*_ = 63.56 years; *SD*_*i*_ = 11.93) were included from the clinical setting. One person was assessed twice by mistake; therefore, second results were excluded. 40.4% were female.

For our first research question we investigated differences in distress between relatives, outpatients, and inpatients. Due to different sample sizing and inconsistent non‐normal distributions, we used Kruskal‐Wallis testing again. We also separated the inpatient group into patients with wish for counseling and without, since all relatives and outpatients implicitly have wished for counseling. There were no differences in overall distress scores (*H*(3) = 5.58, *p* = 0.13). Medians show slightly higher scores for relatives and inpatients with wish for counseling (*M*_*r*_ = 6.60 vs. *M*_*o*_ = 6.31 and *M*_iC_ = 7.29 vs. *M*_iNC_ = 5.81).

For psychosocial distress there were significant differences (*H*(3) = 18.77, *p* < 0.001) between groups. Inpatients without counseling wish showed lowest psychosocial distress (*M*_iNC_ = 0.70 vs. *M*_*r*_ = 1.50 and *M*_*o*_ = 2.00) compared to outpatients (*z* = 4.08; *p* < 0.001; *r* = 0.43) and relatives (*z* = 2.53; *p* = 0.01; *r* = 0.28) but not compared to inpatients with counseling wish (*z* = −1.00; *p* = 0.32; *r* = 0.11). Inpatients with counseling wish did not differ from relatives (*z* = 1.19; *p* = 0.23; *r* = 0.19), but showed lower psychosocial distress than outpatients (*M*_iC_ = 1.20; *z* = 2.23; *p* = 0.03; *r* = 0.32). There were no differences between outpatients and relatives (*z* = −0.98; *p* = 0.33).

Psychological distress also differed between both patient groups and relatives (*H*(3) = 20.76, *p* < 0.001). Inpatients without wish for counseling (*M*_iNC_ = 3.50) showed lower psychological distress compared to relatives (*M*_*r*_ = 5.70; *z* = 2.60; *p* = 0.01; *r* = 0.28) and outpatients (*M*_*o*_ = 6.60; *z* = 4.16; *p* < 0.001; *r* = 0.43). Inpatients with wish for counseling (*M*_iC_ = 5.40) differed from inpatients (*z* = −2.3; *p* = 0.02; *r* = 0.25) but not relatives (*z* = 0.25; *p* = 0.81) or outpatients (*z* = 1.24; *p* = 0.22). No differences were found between outpatients and relatives (*z* = −0.97; *p* = 0.33).

**FIGURE 2:**
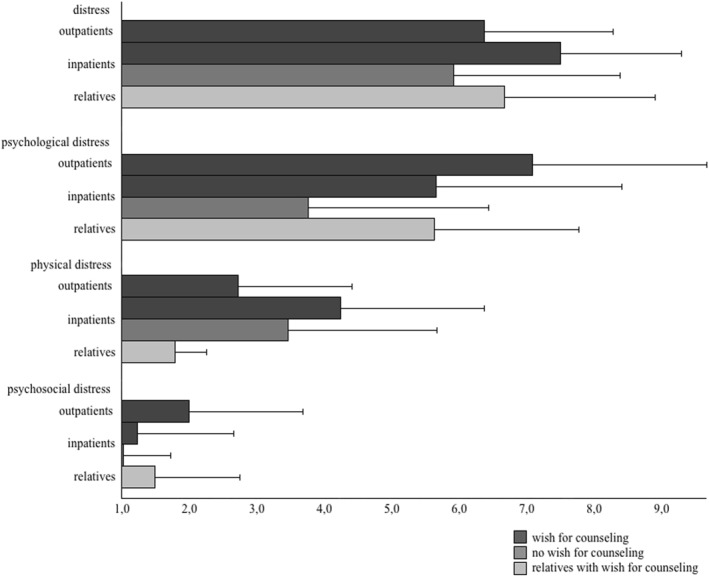
Distress means for each patient subgroup. Distress subscales transformed for comparability. Significant results not shown graphically for a clearer display.

There were also differences in physical distress (*H*(3) = 25.35; *p* < 0.001). Inpatients without wish for counseling (*M*_iNC_ = 3.70) showed significantly more physical distress than relatives (*M*_*r*_ = 1.80; *z* = −4.01; *p* < 0.001; *r* = 0.47) and outpatients (*M*_*o*_ = 2.70; *z* = −2.15; *p* = 0.03; *r* = 0.24), as well as inpatients with wish for counseling (*M*_iC_ = 4.30) also showed more physical distress than relatives (*z* = −4.39; *p* < 0.001; *r* = 0.67) and outpatients (*z* = −2.84; *p* = 0.004; *r* = 0.41) but not inpatients without wish for counseling (*z* = −1.29; *p* = 0.197). See Figure 2 and Table 1 for a clearer display.

For our second research question, we used non‐parametric Kruskal‐Wallis test due to inconsistent non‐normal distribution among variables (*p* > 0.05 for Kolmogorov‐Smirnov test). Analysis showed a significant difference in usability between analogue screening and digital text screening (*H*(2) = 6.7, *p* = 0.035). Post‐hoc Dunn‐Bonferroni test showed only analogue and digital text screenings differ (*z* = −2.38; *p* = 0.017) but not digital video screening (*z* = 1.94 und *p* = 0.05). With *r* = 0.22 there is a small effect size. Digital screening showed higher usability than analogue screening (*M*_*1*_ = 8.87 vs. *M*_*0*_ = 8.40). It is noticeable though that digital video screening shows almost identical mean ranks (*M*_*1*_ = 67.88 vs. *M*_*2*_ = 67.89) but only half of the population (*n* = 30). With *n* < 40 asymptotical significance is too inaccurate and explicit statistics (*p* = 1.0 und *z* = 0) with digital text screening, it is to be assumed, that with exact significance or equal sample size and distribution there would be a significant difference.

**FIGURE 3:**
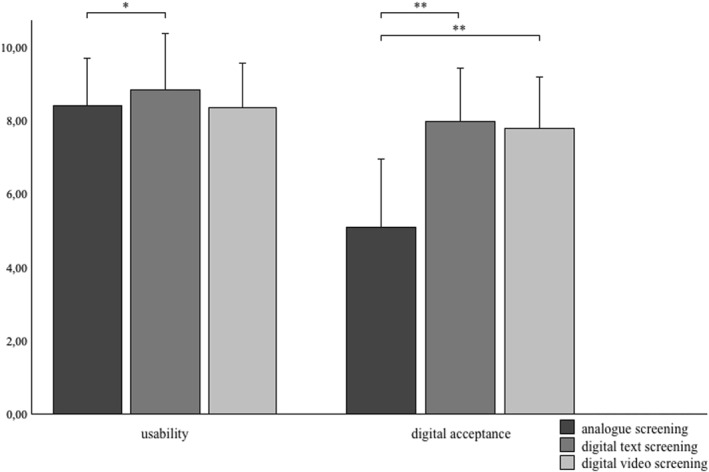
Usability and digital acceptance means for each screening method. *p < 0.05; **p < 0.001.

Digital acceptance was lower for participants provided with analogue screening (*M*_*0*_ = 4.83; *H*(2) = 40.99, *p* < 0.001), compared to digital text (*M*_*1*_ = 7.61; *z* = −5.99; *p* < 0.001) and digital video screening (*M*_*2*_ = 7.43; *z* = −4.47; *p* < 0.001). Effect size was *r* = 0.55 und *r* = 0.47 and showed a medium effect. Especially relatives (*H*(1) = 4.22, *p* = 0.04) and outpatients (*H*(1) = 4.67, *p* = 0.03) showed significant differences in usability ratings, while outpatients (*H*(1) = 20.45, *p* < 0.001) and inpatients (*H*(2) = 20.86, *p* < 0.001) showed differences in digital acceptance between analogue and digital screening. Digital video screening, which was only assessed in inpatients, showed no significant differences for the comparison of digital text versus video screening. See Figure 3 and Table 2 for a clearer display.

We also aimed to explore the relationships between variables such as age or gender with distress, digital acceptance, and usability (see Table 3). Since distribution, variables and study design were sadly not ideal for regression analysis, we used Spearman's correlation. Age was significantly correlated with psychosocial (*r* = −0.45**), psychological (*r* = −0.35**), physical distress (*r* = 0.01), usability (*r* = −0.12), and digital acceptance (*r* = −0.28**). Gender was correlating with psychosocial (*r* = 0.14**), psychological (*r* = 0.17*), physical distress (*r* = −0.12), usability (*r* = 0.12), and digital acceptance (*r* = −0.11). Wish for counseling was correlating with psychosocial (*r* = 0.33**), psychological (*r* = 0.38**), and physical distress (*r* = −0.22*). No harms were reported.

**TABLE 3:** Correlations of distress subtypes, usability, and digital acceptance.

	Age	Gender
Psychosocial distress	−0.45^**^	0.14^**^
Psychological distress	−0.35^**^	0.17^*^
Physical distress	0.01	−0.12
Usability	−0.12	0.12
Digital acceptance	−0.28^**^	−0.11

^*^ *p* < 0.05.

^**^ *p* < 0.001.

## 4 Discussion

This study examined the disparities in distress experienced by outpatients and inpatients with cancer as well as their relatives. In order to advance digitalisation in future medicine, we also investigated the acceptability and usability of digital screening approaches.

In our study, overall distress levels on average were moderate to high in all groups, confirming results of previous research. There were no significant differences between relatives and patient groups, though, distress dimensions varied slightly. Outpatients and relatives experienced significantly more psychosocial and psychological distress than inpatients without wish for counseling. Inpatients with wish for counseling had distress levels similar to outpatients and relatives with no significant differences. Inpatient groups showed higher physical distress.

More recent research on psychological coping during active medical treatments showed that psychological defense mechanisms (e.g., suppression, denial) can be highly active and limit access to current emotional and psychological states. Psychological distress often becomes more prevalent after the end of treatment [31, 32, 33], which would be in accordance with our findings in outpatients. For relatives, having hospitalised family members is causing distress, however, compensating care work at home is leading to sometimes even greater distress for care takers [30].

During hospitalisation, though hospitalisation effect can increase distress [34], patients receive daily support from hospital staff and visitations, while outpatients have limited contacts. In remission, cancer patients often feel abandoned due to their social environment's normalcy, while survivor's normalcy is still a long way to go (hot potato effect) [9, 12]. Both outpatients and inpatients have different distress factors, which can explain the heterogeneous research findings. This highlights the importance of counseling and psychotherapy for cancer patients, regardless of their physical condition or hospitalisation status.

The desire for counseling appeared to be a significant factor, given the substantial differences between inpatients without and with explicit wish for counseling, as well as the strong correlations observed. Patients and relatives who expressed a desire for counseling tended to experience higher levels of distress and should therefore receive support. Unfortunately, this is still far from reality [3, 35].

Correlations support current research about higher distress among younger patients [36] while gender had a smaller effect than expected. This can easily be explained for the numerous challenges AYA cancer patients have to endure like long term infertility, taking care of younger children, financial instability, non‐completed education and instable employment, feeling of missing out on active lifestyle and pressure from relatives and friends.

Usability and digital acceptance received moderate to high ratings. Digital text‐based screening ranked highest in acceptance and usability, with video screening following closely behind. This suggests that digital screening should be prioritised over analogue methods in the future. However, additional expenses for creating instructional videos may be unnecessary, as digital text‐based instruction was slightly superior. Participants in the digital text screening group gave particularly high ratings for acceptance and usability. Relatives and outpatients gave slightly higher usability and digital acceptance ratings. Younger and male participants showed slightly higher acceptance and usability scores. These findings suggest, regardless of age or gender, acceptance of digital screening tools improves once individuals have hands‐on experiences.

As previously mentioned, digital screening shows superior advantages compared to analogue screening. Our medical staff was visibly relieved by the digitalisation due to time saving, as printing and patient instruction was shortened and led to faster evaluation. Staff could directly see evaluated results in the respective already existing digital patient file and act accordingly to high distress levels or expressed wish for counseling. One particular advantage was reduced infection risk during COVID‐19 pandemic, which stays relevant for immunocompromised cancer patients even after the pandemic. There were noticeable fewer missing items, as digital screening is able to use forced responses. We also could adjust the screen to the different needs of each participant, for instance font size, screen brightness, and contrast. Participants with fatigue, impaired joints or inability to hold pencils, could simply tab their answers, after staff placed the tablet correctly and accessibly.

Digital screening methods have their own disadvantages, albeit different ones than analogue technology. One of the main concerns for many clinics is the constant maintenance of hardware and software. Exchanging hardware for newer technology can be expensive, as well as time consuming to update software and maintain compatibility. Screening for outpatients outside of clinic environment can be challenging due to variability in hardware and uncontrolled test environments [37].

Another concern is the accessibility and skilled handling of technology. Especially older patients may feel discouraged to complete screenings. In our trials we were unable to confirm this assumption, as all elderly patients demonstrated sufficient technological skills or required only minimal assistance and encouragement. It can be assumed that this assistance will continue to decrease with subsequent generations. The last major concerns regarding digital screening are the accessibility for impaired patients, such as impaired vision or speech, as well as data storage. Both can be addressed with the use of appropriate technology and customisable settings. Our elderly patients with impaired vision could use settings to customise font size and contrast. Data storage was implemented in our already existing digital patient files, leading to no additional workload for staff.

Apart from the disadvantages for digital text‐based screening mentioned so far, there are further disadvantages for digital video‐based screening. The major difficulty is to create an understandable and accessible video for every patient. While digital text can be adjusted easily, the video can't be customised to every need. While screen brightness and zoom could be adjusted, some patients expressed the need for adjusting the playback speed. Without that certain setting, videos had to be paused and rewind, leading to higher frustration.

### 4.1 Limitations

This study aimed at comparing distress levels of different patient groups, while testing the feasibility of introducing digital screenings versus established analogue screenings to assess distress. In a later project phase we had the chance to expand the study with including inpatients, as well as a digital video‐based screening. Due to copyright reasons of the video platform and delayed inclusion of inpatients, this lead to missing subgroups of outpatients or relatives with digital screening with video‐based instruction. This limited our chances for comparing digital to analogue screening in outpatients or relatives to inpatients.

In our outpatient and relatives' samples we naturally had a selective sample of participants who actively sought out psychological counseling. This could have enhanced the effect of higher distress in those groups. We therefore separated inpatients by their wish for counseling, which lead to uneven group sizing. Future research should verify results, with matched substantial group differences.

One reason for improved acceptance of digital screening was the timeline during the COVID‐19 pandemic, which led to increased use of digital applications. Similarly, the timeline of COVID‐19 could have led to an increased level of distress. Most cancer patients are immunosuppressed and therefore anxious to catch an infection, as well as reduced hospital visits and less support services.

### 4.2 Conclusion

Our study provides valuable insights in distress levels of different patient subgroups, such as inpatients, outpatients, and relatives. Furthermore, we could show convincing usability and acceptance of digital screening tools. Outpatients experienced comparable or even higher psychosocial and psychological distress, compared to inpatients showing higher physical distress. In this study, the effect is limited to inpatients without wish for counseling. Inpatients with wish for counseling showed similar psychosocial and psychological distress to outpatients and relatives.

Digital distress screening is widely accepted by cancer patients and relatives, offering a standardised, practical, and economical approach for education, instruction, and screening distress. In this study, patients of high age as well as a small number of women may prefer analogue screening, however, with hands‐on experiences of digital tools, acceptance rates increased strongly. Other findings support no differences between gender or age in terms of digital acceptance. Both digital text and video instructions are well‐accepted, and can be implemented depending on resources and preferences within clinical departments. Instruction by video did not show beneficial effects.

### 4.3 Implications

Psycho‐oncological services are increasingly being established in hospitals to support patients and their families during cancer treatment. As outpatient care has grown, it is crucial to include outpatients but also relatives of cancer patients in research, as our study shows high distress scores. It seems to be crucial to pay attention to the different types of distress in each patient group. Distress screening should be standard procedure for inpatients with repeated measurements, outpatients during day care treatment and remission check‐ups, as well as for relatives in counseling centres. To express wish for counseling should be encouraged, leading to receive counseling options, due to higher symptom severity. Our findings support the digitalisation of screening tools, with less workload for staff and high acceptance by patients. The most important step seems to be the hands‐on experience, especially for more skeptical patient subgroups, such as elderly patients. Contrary to our expectations, we experienced only minimal need of assistance or encouragement. The extra step of creating animated videos seems unnecessary. Cancer centres should consider their technological resources and act accordingly to establish digital screening tools. While not everyone has the resources to program their own app, there are other options that are easier to develop and require less maintenance, making digital screening available for every cancer treatment centre.

## Author Contributions

**Tana Dornbrach:** conceptualization, investigation, software, data management, data analysis, writing – original, revision and editing. **Madeleine Volz:** conceptualization, investigation, data collection and management, writing – original, validation. **Thomas Seufferlein:** investigation process, resource provision. **Hans Kestler:** conceptualization, investigation, resource provision, funding acquisition, project administration. **Klaus Hönig:** conceptualization, investigation, data analysis, resource provision, funding acquisition, project administration, supervision, validation, manuscript revision and editing.

## Funding

This research was supported by the Germany Federal Ministry of Education and Research (BMBF) as part of the DIFUTURE project (Medical Informatics Initiative, Grant No. 01ZZ1804I), from the Ministry of Science and Art Baden‐Württemberg (Zentrum für Innovative Versorgung, ZIV) and from the Ministry of Social Affairs of the state of Baden Württemberg, Germany (projects: feelback and ZPM).

## Ethics Statement

This study was performed in line with the principles of the Declaration of Helsinki. Approval was granted by the Research Ethics Committee of the University of Ulm (protocol code 136/21 of 14.04.2021). Study design and used materials were conducted in accordance with the local legislation and institutional requirements. Written informed consent was required by all participants.

## Conflicts of Interest

The authors declare no conflicts of interest.

## Study Registration

This study was registered at the Ulm Trial Management System of Comprehensive Cancer Center Ulm (CCCU) with the ID 10505 at 1st of April 2020.

## Patient Participation

This paper was generated with active participation of cancer patients.

## Data Availability

The data that support the findings of this study are available on request from the corresponding author. The data are not publicly available due to privacy or ethical restrictions.

## Tables

**TABLE A1:** Demographic and characteristics of relatives and patients.

	Relatives (*n* = 25)	Outpatients (*n* = 35)	Inpatients (*n* = 89)
Analogue screening	13 (52%)	18 (51.4%)	31 (34.8%)
Digital text screening	12 (48%)	17 (48.6%)	28 (31.5%)
Digital video screening	0 (0%)	0 (0%)	30 (33.7%)
Age *M* ± SD (range)	49.04 ± 14.80 (23–77)	56.54 ± 10.32 (26–77)	63.56 ± 11.93 (27–86)
Gender			
Male	7 (28%)	8 (22.9%)	53 (59.6%)
Female	18 (72%)	27 (77.1%)	36 (40.4%)
Relationship			
Single	4 (16%)	3 (8.5%)	6 (6.7%)
In partnership	2 (8%)	5 (14.3%)	5 (5.6%)
Married	14 (56%)	25 (71.4%)	63 (70.8%)
Divorced	1 (4%)	1 (2.9%)	8 (9%)
Widowed	4 (16%)	1 (2.9%)	7 (7.9%)
Children			
Yes	11 (44%)	27 (71.1%)	78 (87.6%)
No	14 (56%)	8 (22.9%)	11 (12.4%)
Nationality			
German	23 (92%)	30 (85.7%)	79 (88.8%)
Other	2 (8%)	5 (14.3%)	7 (7.9%)
Level of education			
Haupt‐/volksschule	2 (8%)	9 (25.7%)	37 (41.6%)
Realschule	8 (32%)	15 (42.9%)	26 (29.2%)
Fachhochschule	4 (16%)	1 (2.9%)	5 (5.6%)
Gymnasium	11 (44%)	10 (28.6%)	18 (20.2%)
Other			3 (3.3%)
Employment status			
Full time	13 (52%)	8 (22.9%)	21 (23.6%)
Part time	7 (28%)	6 (17.1%)	26 (29.2%)
No, pension	3 (12%)	10 (28.6%)	50 (56.2%)
No, other	2 (8%)	11 (31.4%)	9 (10.1%)
Sick leave			
Yes	3 (12%)	14 (40%)	27 (30.3%)
No	22 (88%)	20 (57.1%)	57 (64.0%)
Cancer diagnosis			
Brain tumour	3 (12%)	3 (8.6%)	0
Mamma carcinoma	1 (4%)	10 (28.6%)	0
Bronchial carcinoma	3 (12%)	2 (5.7%)	2 (2.2%)
Pancreatic carcinoma	2 (8%)	1 (2.9%)	26 (29.2%)
Colorectal/duodenal carcinoma	0	2 (5.7%)	18 (20.2%)
Haematological cancer	3 (12%)	6 (17.1%)	1 (1.1%)
Testicle/prostate carcinoma	1 (4%)	3 (8.6%)	1 (1.1%)
Gynaecological cancer	2 (8%)	2 (5.7%)	1 (1.1%)
Adenocarcinoma	0	3 (8.6%)	1 (1.1%)
Oesophageal carcinoma	0	0	8 (9.0%)
Gastric carcinoma	0	0	12 (13.5%)
Hepatocellular/renal carcinoma	2 (8%)	0	13 (14.6%)
Malignant melanoma/squamous cell carcinoma	1 (4%)	0	0
Sarcoma	1 (4%)	0	0
Other	2 (8%)	2 (5.7%)	6 (6.7%)
Wish for counseling			
Yes	25 (100%)	35 (100%)	21 (23.6%)
No	0 (0%)	0 (0%)	68 (76.4%)
Distress thermometer			
*M* ± SD (range)	6.60 ± 2.35 (2–10)	6.31 ± 2.03 (2–10)	6.16 ± 2.56 (0–10)
Psychosocial distress	1.50 ± 1.30	2.00 ± 1.70	0.80 ± 1.20
Psychological distress	5.70 ± 2.30	6.60 ± 3.30	4.00 ± 3.10
Physical distress	1.80 ± 1.50	2.70 ± 1.60	3.90 ± 2.00
Usability	8.90 ± 1.01	8.70 ± 1.20	8.40 ± 1.53
Digital acceptance	6.93 ± 1.86	6.21 ± 2.68	6.37 ± 2.81

**TABLE A2:** McDonald's omega of all scales.

Scale	McDonald's *ω* All subjects	McDonald's *ω* Outpatients	McDonald's *ω* Inpatients
Psychosocial distress	0.54	0.51	0.51
Psychological distress	0.76	0.71	0.75
Physical distress	0.81	0.79	0.78
Usability	0.62	0.48	0.66
Digital acceptance	0.91	0.86	0.93
